# Deep learning-based histopathological classification and subclassification of benign and malignant salivary gland tumors

**DOI:** 10.1007/s00405-026-10082-6

**Published:** 2026-03-05

**Authors:** Andreas Weber, Daniel Schuster, Jannis Heyer, Christoph Becker, Valentin Burkhardt, Martin Werner, Andreas Spörlein, Peter Bronsert, Tobias Schulz

**Affiliations:** 1 Institute for Surgical Pathology, Medical Center, Faculty of Medicine, University of Freiburg, Freiburg, Germany; 2 Faculty of Biology, University of Freiburg, Freiburg, Germany; 3 Department of Oto-Rhino-Laryngology, Medical Center, Faculty of Medicine, University of Freiburg, Freiburg, Germany; 4 Core Facility for Histopathology and Digital Pathology, Medical Center, University of Freiburg, Freiburg, Germany; 5 Biobank Comprehensive Cancer Center Freiburg, Medical Center, University of Freiburg, Freiburg, Germany; 6 Department of Oto-Rhino-Laryngology, Medical Center, Faculty of Medicine, University of Freiburg, Killianstrasse 5, Freiburg, 79106 Germany

**Keywords:** Machine learning, artificial intelligence, automated diagnostics, digital pathology, salivary gland tumors, deep learning, convolutional neural networks

## Abstract

**Background:**

Salivary gland tumors are rare and histopathologically diverse, presenting a diagnostic challenge. Accurate diagnosis is essential for optimal treatment planning. This study evaluates deep learning models in distinguishing benign from malignant salivary gland tumors using hematoxylin and eosin-stained histopathological images.

**Methods:**

184 patients with the diagnosis of a salivary gland tumor (131 benign, 53 malignant) and six patients with normal salivary gland tissue were included. In total 335 histological sections were digitized. Deep learning models - including VGG19, ResNet50, Inception-ResNet-v2, Xception, ConvNeXt, and a Vision Transformer - were trained to classify benign tumors (Warthin tumor, pleomorphic adenoma) from malignant tumors (mucoepidermoid carcinoma, acinic cell carcinoma, adenoid cystic carcinoma, squamous cell carcinoma). The models’ performance in sub classifying benign tumors and differentiating them from normal tissue was also examined, as well as classification among the malignant tumors and normal tissue.

**Results:**

All models showed strong performance in classifying benign versus malignant tumors, with the Xception model achieving 100% balanced accuracy. Corresponding confusion matrices indicated nearly error-free differentiation. Subclassification of the four malignant tumors against each other and normal tissue yielded balanced accuracies between 56.45% and 71.51%. Class activation maps revealed the models focused on stromal components in benign tumors and on cell nuclei and cytoplasm in malignant tumors.

**Conclusion:**

Deep learning models show great potential for the automated classification of salivary gland tumors as benign or malignant. Our findings suggest that deep learning models could be a valuable tool in clinical practice, particularly in the differentiation between benign and malignant lesions. However, limitations in malignant subtyping and the restricted number of malignant cases warrant further validation in larger, multi-institutional datasets.

**Supplementary Information:**

The online version contains supplementary material available at 10.1007/s00405-026-10082-6.

## Introduction

With an approximated incidence of 2.5-3.0 per 100,000 inhabitants, salivary gland tumors are a relatively rare tumor entity in Western nations [1]. The parotid gland represents the most frequent localization for benign tumors (70%), while the minor glands are the most prevalent location for malignant tumors [1].

According to the latest WHO classification from 2022, there are 15 benign epithelial and 21 malignant epithelial entities of salivary gland tumors [2].

The histopathological variety, together with the rarity of these tumors, represents a significant diagnostic challenge in histopathological practice. Initial hematoxylin and eosin (H&E) based evaluation and subsequent immunohistochemical staining for final diagnosis can be time and cost intensive.

Depending on the tumor entity (benign / malignant), a revision surgery may be indicated. This surgical procedure should be performed promptly, since the facial nerve becomes anatomically more difficult to identify with increasing scarring in the surgical area, resulting in an increased risk of postoperative facial nerve palsy [3, 4].

The aim of the study is to investigate the performance of Convolutional Neural Networks (CNNs) and Vision Transformer (ViT) in automated histopathological diagnosis of benign and malignant salivary gland tumors. Therefore, deep learning (DL) models classifying benign (pleomorphic adenoma and Warthin tumor (cystadenolymphoma)) and epithelial malignant salivary gland tumors (adenoid cystic carcinoma, mucoepidermoid carcinoma, acinic cell carcinoma, and squamous cell carcinoma) using H&E-stained histopathology slides are employed. Furthermore, class activation maps and attention maps are used to visualize the key regions of the input images contributing most to a model’s prediction.

## Methods

### Ethical approval

All human studies and research on human tissue described in this paper were carried out with the approval of the relevant ethics committee, in accordance with national laws and the revised version of the 1975 Declaration of Helsinki. The ethical guidelines stated in each case apply to the listed studies. The research project was reviewed and approved by the ethics committee (Ethics committee submission number 167/18). All patients whose histological sections were included in the study demonstrated their acceptance of an informed written consent form, which was approved by the relevant ethics committee.

### Study protocol and sample acquisition

A total of 53 patients with a diagnosis of malignant salivary gland tumor, 131 patients with a diagnosis of benign salivary gland tumor and six patients with normal salivary gland tissue were included into the presented study. In terms of benign tumors, 61 patients had pleomorphic adenoma and 70 patients had Warthin tumor. Among the malignant tumors, 11 patients had mucoepidermoid carcinoma, 13 patients had acinic cell carcinoma, 12 patients had adenoid cystic carcinoma. 17 patients had squamous cell carcinoma (including four metastases from a primary squamous cutaneous carcinoma). Only tumor entities with histological slides from at least three patients were included. All patients underwent surgery between January 2003 and December 2023 at the Department of Otolaryngology at the University Hospital Freiburg, Germany. All tissue specimens were processed and diagnosed at the Institute of Clinical Pathology Freiburg, Germany.

### Image acquisition

The histopathological slides were digitized at high resolution using a magnification of x40 (PANNORAMIC 1000, 3DHistech Kft., H-1141 Budapest, Öv u., Hungary). In total 240 whole slide images (WSIs) of benign tumors (115 images of pleomorphic adenoma and 125 images of Warthin tumor), 87 WSIs of malignant tumors (19 mucoepidermoid carcinoma, 22 acinic cell carcinoma, 20 adenoid cystic carcinoma, 26 squamous cell carcinoma) and eight WSIs, featuring only normal salivary gland tissue were included into the presented study.

### Histopathological evaluation

Tumor tissue and adjacent normal salivary gland tissue were manually annotated using QuPath under supervision of surgical pathology residents and fellows [5]. A total of 1,227 annotations as pleomorphic adenoma, 2,481 as Warthin tumor, 2,704 as mucoepidermoid carcinoma, 2,382 as acinic cell carcinoma, 2,448 as adenoid cystic carcinoma, 3,203 as squamous cell carcinoma, and 4,635 as normal salivary gland tissue were obtained.

### Data set generation

Each image was normalized to the pixel distribution according to Reinhard et al. [6]. Pixel values were converted from RGB to Lab color space and the distributions in the a and b channels of a target image were matched to those of the source image. Following normalization, each image was split into tiles of 250 × 250 pixels. A tile was considered as labeled if at least 99% of its area overlapped with the corresponding annotation. The dataset was split into a training, validation and test set, with approximately 80% used data for the training and validation set and approximately 20% used data for testing.

### Data split for classification of benign and malignant tumors

Data split into training, validation and test set was performed on the image level. Due to hardware restrictions, 218 images from 125 patients were randomly selected. This yielded 640,139 tiles labeled as “benign” and 487,119 tiles labeled as “malignant”. Benign tiles include Warthin tumor and pleomorphic adenoma. Malignant tiles include squamous cell carcinoma, acinic cell carcinoma, adenoid cystic carcinoma and mucoepidermoid carcinoma (Figure S1 demonstrating exemplary tiles for each class label and Figure S2 demonstrating the class distribution for training, validation and test set).

### Data split for subclassification of benign tissues

The data set for the subclassification of benign tissues comprised 240 images from 131 patients yielding 54,496 tiles labeled as “salivary gland”, 198,875 tiles labeled as “Warthin tumor” and 1,158,413 tiles labeled as “pleomorphic adenoma” (Figure S3) Due to sufficient number of patients, the split into training, validation and test set was performed on patient level. Figure S4 shows the class distribution for the training, validation and test sets.

### Data split for classification of malignant and normal tissue and subclassification of malignant tissues

The data set comprised 95 images (87 malignant tissue and 8 normal salivary gland tissue) from 53 patients yielding 70,679 tiles labeled as “squamous cell carcinoma”, 339,592 tiles labeled as “acinic cell carcinoma”, 361,661 tiles labeled as “adenoid cystic carcinoma” 139,527 tiles labeled as “mucoepidermoid carcinoma” as well as 52,615 tiles labeled as “salivary gland tissue” (Figure S5). The data was split into training, validation and test sets on image level with approximately 80% allocated to the training and validation set and 20% used for the test set (Figure S6).

### Deep learning-based evaluation of images

In order to find an optimal DL-algorithm for the given task, we compared the performances of VGG19 [7], ResNet50 [8], Inception-ResNet-v2 [9], Xception [10], ConvNeXt-L [11] and Vision Transformer (ViT) [12]. A learning rate of 0.0001 and batch size of 30 were selected. For the classification of benign and malignant and the subclassification of benign tissue, all networks were trained for 5 epochs. For the subclassification of malignant tissue, all networks were trained for 8 epochs. All computations were performed using Python 3.9.16 and Tensorflow 2.6.0 [13] on an NVIDIA Geforce RTX 4090.

### Performance evaluation of deep learning algorithms

The metrics precision, recall and F1-score [14] for each class on the test set and the overall balanced accuracy on the test set were reported. Additionally, confusion matrices were presented, showing the number of predicted and actual tiles for the test set.

### Class activation maps and attention maps

Class activation maps (CAMs) [15] for CNNs are generated by leveraging global average pooling (GAP) in the final convolutional layer. GAP reduces the spatial dimensions of each feature map to a single value, reflecting the importance of that feature map in the network’s final prediction. CAMs reflect the contribution of different image regions to the network’s output, thus providing a spatial map of the areas that influenced the decision. For Vision Transformers, attention maps are used to visualize the regions of the image where the network focuses its attention. The ViT architecture employs multi-head attention, where multiple sets of attention weights are learned. The attention maps are derived by aggregating max fusion information across these multiple attention heads, providing a view of the areas within the image that are most influential in the network’s prediction.

## Results

### Classification of benign and malignant tumors

The binary classification between benign and malignant tumors yielded almost perfect results across most network architectures. In detail, Xception performed best with a balanced accuracy score of 100% (rounded to the fourth decimal place). More performance metrics are shown in Table 1. Confusion matrices are shown in the supplements Figure S7.

**Table 1:** Performance metrics for classification benign and malignant tumors on the test set

Network	Class	Precision	Recall	F1-Score	Balanced Accuracy
VGG19	benign	1.0000	0.9993	0.9996	0.9997
	malignant	0.9991	1.0000	0.9995	
ResNet50	benign	1.0000	0.8258	0.9046	0.9129
	malignant	0.8149	1.0000	0.8980	
Inception-ResNet-v2	benign	0.9984	0.9993	0.9988	0.9986
	malignant	0.9991	0.9979	0.9985	
Xception	benign	1.0000	1.0000	1.0000	1.0000
	malignant	1.0000	1.0000	1.0000	
ConvNeXt	benign	0.9991	0.9982	0.9986	0.9985
	malignant	0.9977	0.9988	0.9982	
Vision-Transformer	benign	0.9993	0.9996	0.9994	0.9993
	malignant	0.9994	0.9991	0.9993	

### Subclassification of benign tissue

The subclassification of benign tissue into normal salivary gland tissue, Warthin tumor and pleomorphic adenoma yielded high performance across all network architectures. Xception performed best with a balanced accuracy score of 95.57%. Detailed performance metrics are shown in Table 2. Confusion matrices are shown in supplements Figure S8.

**Table 2:** Performance metrics for subclassification of benign tissue into normal salivary gland tissue (nSGT), Warthin tumor (WT) and pleomorphic adenoma (PA) on the test set

Network	Class	Precision	Recall	F1-Score	Balanced Accuracy
VGG19	nSGT	0.6596	0.9415	0.7757	0.9222
	WT	0.6923	0.9205	0.7903	
	PA	0.9837	0.9046	0.9425	
ResNet50	nSGT	0.5645	0.9621	0.7116	0.9483
	WT	0.9231	0.9269	0.9250	
	PA	0.9905	0.9557	0.9728	
Inception-ResNet-v2	nSGT	0.5650	0.9537	0.7096	0.9172
	WT	0.6172	0.9354	0.7437	
	PA	0.9879	0.8627	0.9210	
Xception	nSGT	0.7430	0.9454	0.8321	0.9557
	WT	0.9296	0.9447	0.9371	
	PA	0.9930	0.9769	0.9848	
ConvNeXt	nSGT	0.7428	0.9568	0.8363	0.9292
	WT	0.8055	0.8833	0.8426	
	PA	0.9782	0.9474	0.9626	
Vision-Transformer	nSGT	0.6717	0.9147	0.7746	0.9329
	WT	0.7820	0.9497	0.8577	
	PA	0.9900	0.9342	0.9613	

### Classification of malignant and normal tissue and subclassification of malignant tissues

The subclassification of malignant tissue into squamous cell carcinoma, acinic cell carcinoma, adenoid cystic carcinoma and mucoepidermoid carcinoma as well as against normal salivary gland tissue yielded suboptimal results. Inception-ResNet-v2 performed best with a balanced accuracy of 71.51%. Detailed performance metrics are shown in Table 3. Confusion matrices are shown in supplements Figure S9.

**Table 3:** Performance metrics for classification of squamous cell carcinoma (SCC), acinic cell carcinoma (AcCC), normal salivary gland tissue (nSGT), adenoid cystic carcinoma (AdCC) and mucoepidermoid carcinoma (MEC) on the test set

Network	Class	Precision	Recall	F1-Score	Balanced Accuracy
VGG19	SCC	0.1985	0.7699	0.3156	0.6833
	AcCC	0.8768	0.2612	0.4025	
	nSGT	0.6682	0.9503	0.7847	
	AdCC	0.6294	0.4891	0.5504	
	MEC	0.5464	0.9457	0.6926	
ResNet50	SCC	0.1521	0.6701	0.2480	0.6265
	AcCC	0.8065	0.2630	0.3967	
	nSGT	0.4394	0.9429	0.5994	
	AdCC	0.6309	0.3576	0.4564	
	MEC	0.5279	0.8987	0.6651	
Inception-ResNet-v2	SCC	0.1611	0.8814	0.2724	0.7151
	AcCC	0.8854	0.2535	0.3941	
	nSGT	0.3076	0.9599	0.4660	
	AdCC	0.8961	0.5629	0.6915	
	MEC	0.6870	0.9180	0.7859	
Xception	SCC	0.1126	0.6604	0.1924	0.6567
	AcCC	0.8593	0.2608	0.4001	
	nSGT	0.4620	0.9618	0.6241	
	AdCC	0.9947	0.5157	0.6793	
	MEC	0.5858	0.8847	0.7049	
ConvNeXt	SCC	0.1063	0.6086	0.1810	0.6078
	AcCC	0.7914	0.2419	0.3705	
	nSGT	0.4199	0.8877	0.5701	
	AdCC	0.9615	0.3959	0.5609	
	MEC	0.5497	0.9058	0.6842	
Vision-Transformer	SCC	0.1002	0.5369	0.1689	0.5645
	AcCC	0.7332	0.2515	0.3745	
	nSGT	0.3620	0.8352	0.5051	
	AdCC	0.9947	0.2957	0.4559	
	MEC	0.5136	0.9033	0.6549	

### Class activation maps

**Fig. 1:**
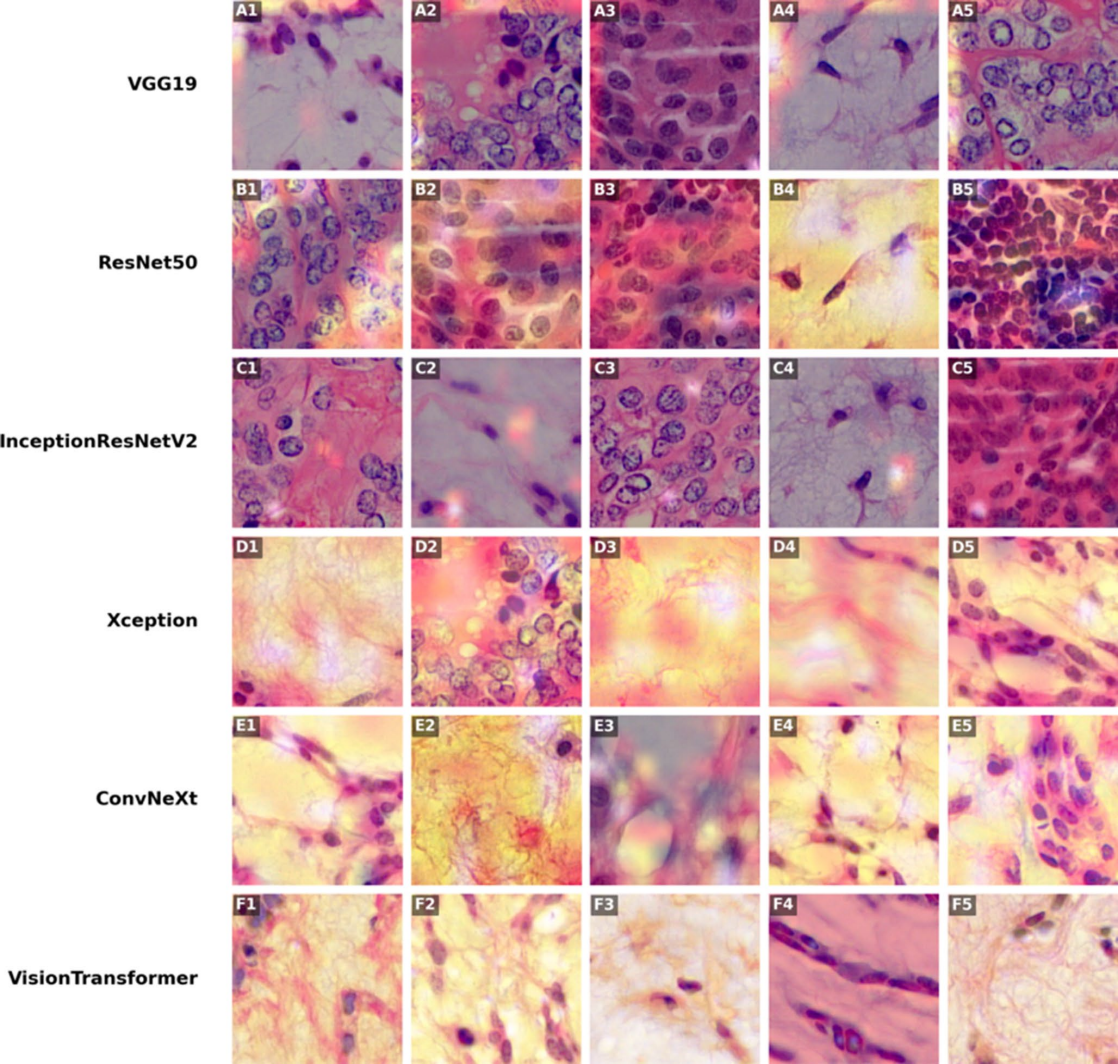
Class activation maps for the correct classification of benign tumors plotted as overlay with the input tile. The colormap of the maps range from black through dark red, orange, yellow, and eventually to white as the importance of the input pixel increases

**Fig. 2:**
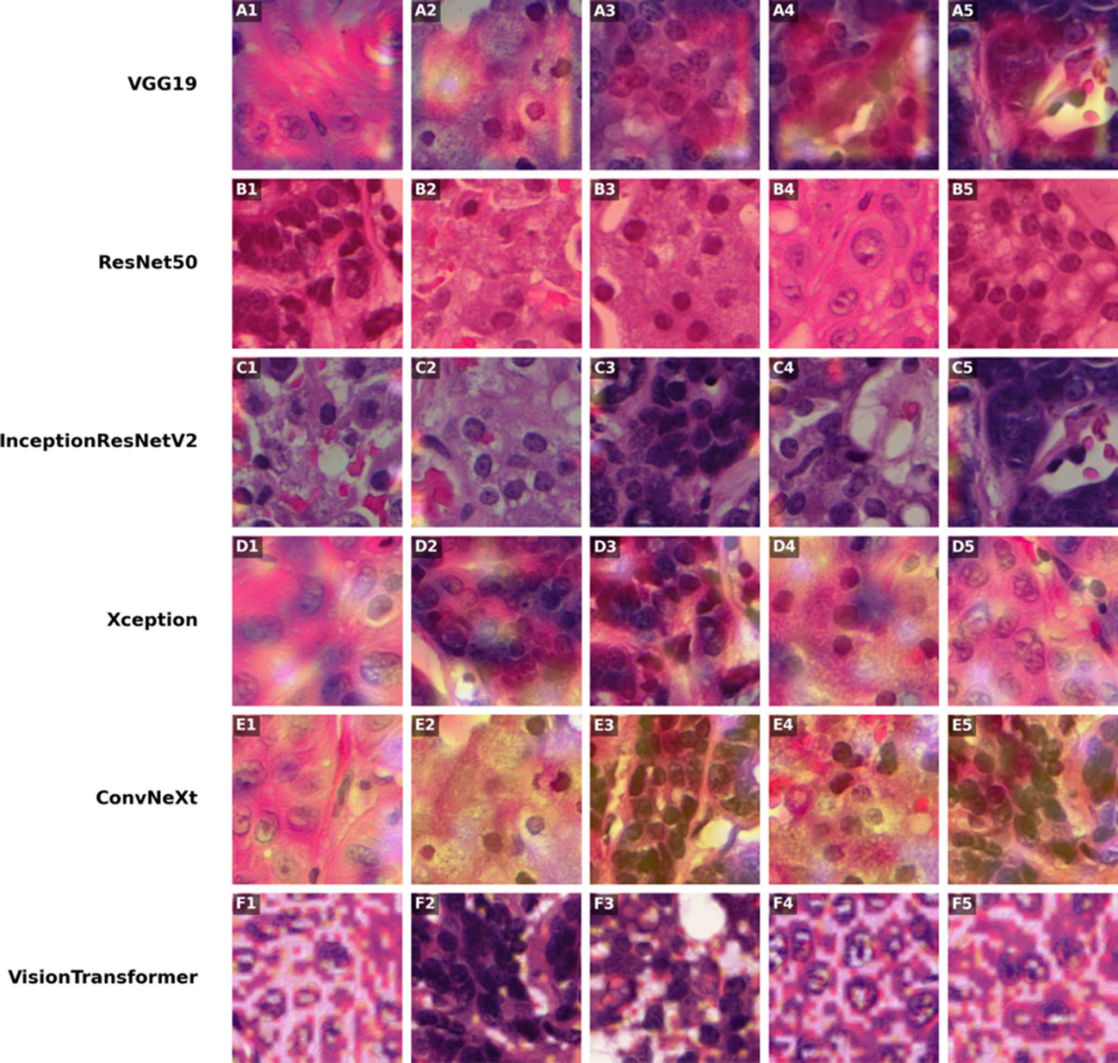
Class activation maps for the correct classification of malignant tissue plotted as overlay with the input tile. The colormap of the maps range from black through dark red, orange, yellow, and eventually to white as the importance of the input pixel increases

**Fig. 3:**
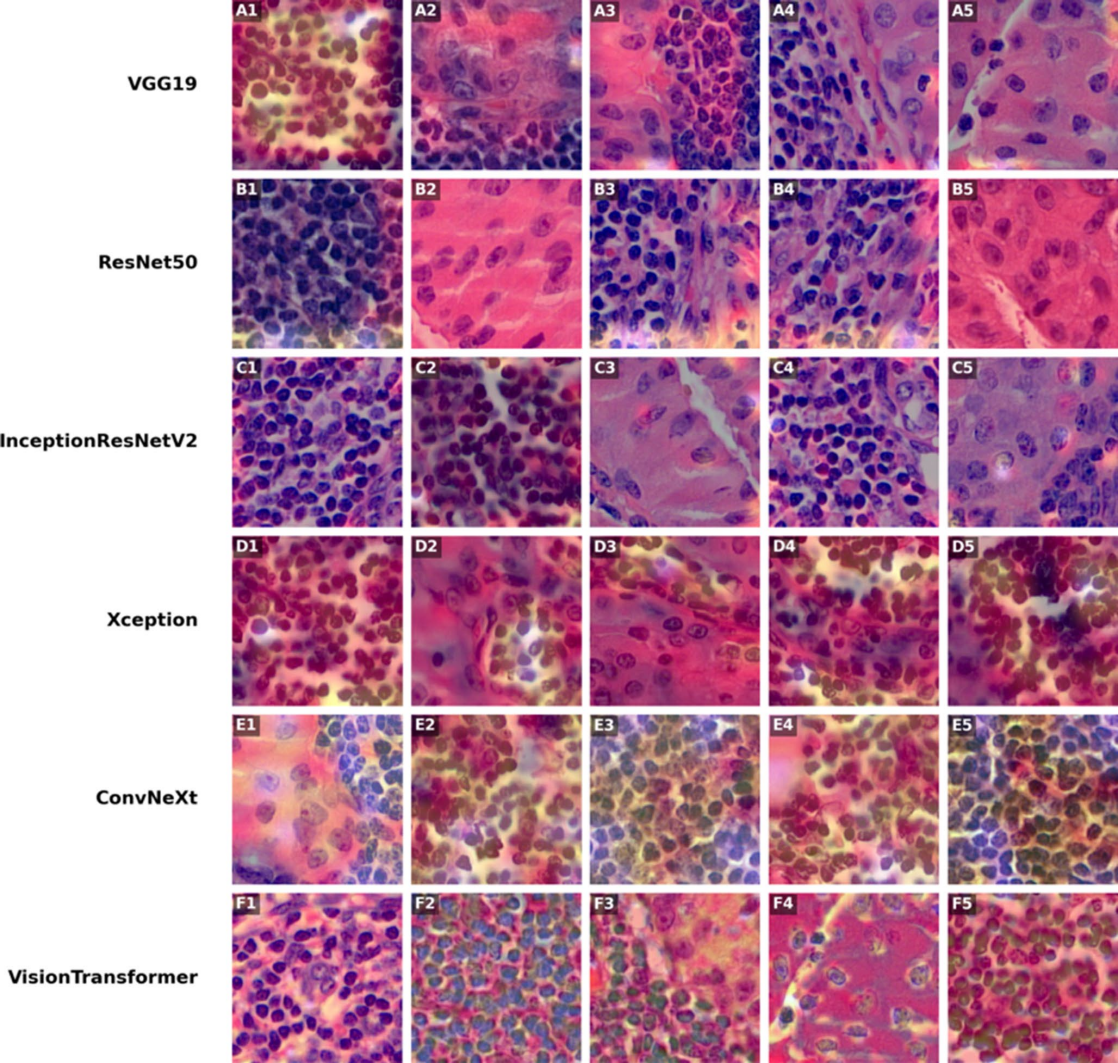
Class activation maps for the correct classification of Warthin tumor plotted as overlay with the input tile. The colormap of the maps range from black through dark red, orange, yellow, and eventually to white as the importance of the input pixel increases

**Fig. 4:**
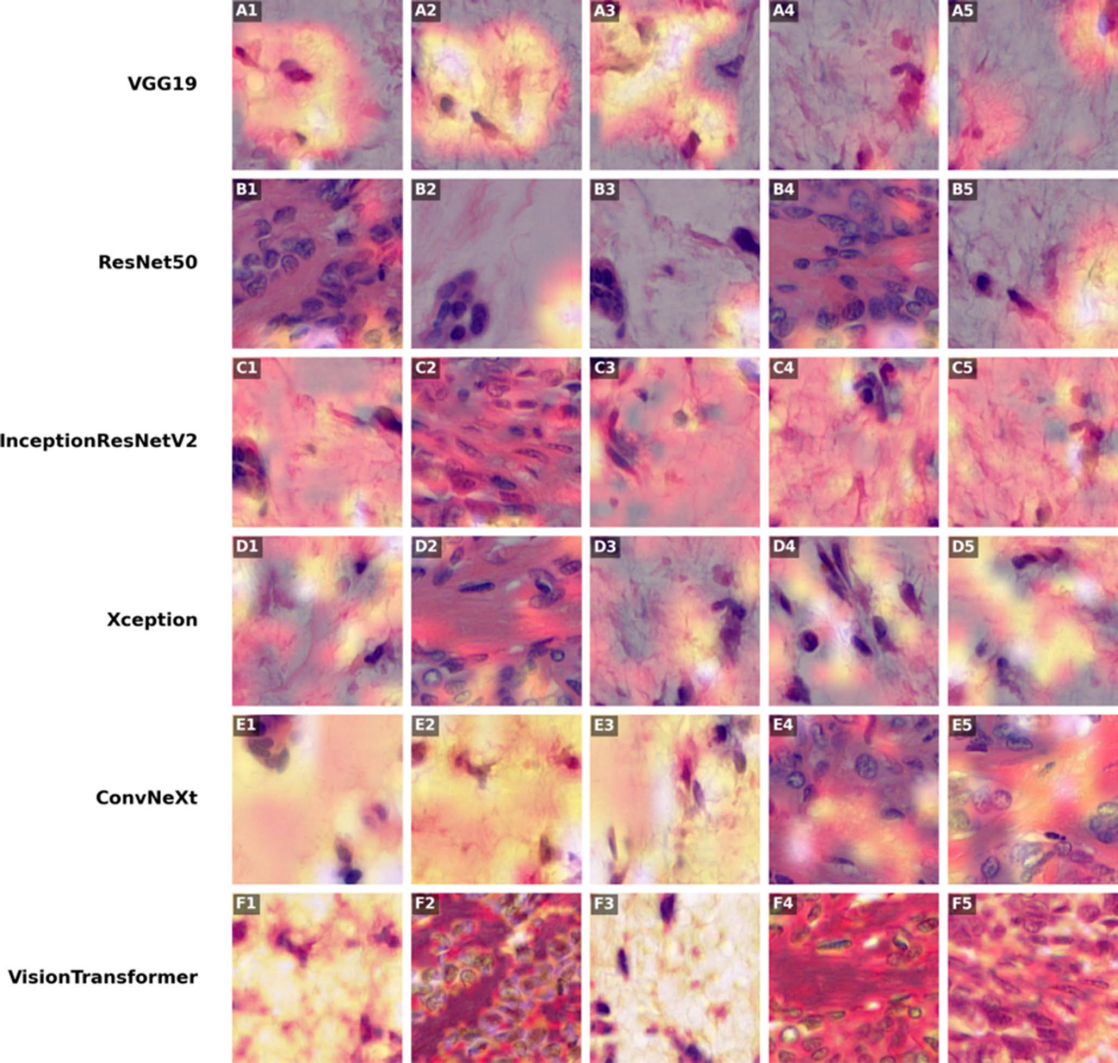
Class activation maps for the correct classification of pleomorphic adenoma plotted as overlay with the input tile. The colormap of the maps range from black through dark red, orange, yellow, and eventually to white as the importance of the input pixel increases

The CAMs for the correct classification of benign tumors indicate that the CNNs prioritized interstitial connective tissue (Fig. 1; B5, D5, E5). In addition, ResNet50 focused on the cytoplasm in terms of cell-rich regions (Fig. 1; B1, B2). To correctly classify malignant tissue, ConvNext (Fig. 2; E3 and E5) and ViT (Fig. 2; F4 and F5) seem to focus rather on cell nuclei. With regard to the correct classification of Warthin tumor, ViT (Fig. 3; F2, F3) and ConvNext (Fig. 3; E3, E5) focused primarily on the lymphoid stroma. Furthermore, ConvNext prioritized the oncocytic epithelium of Warthin tumor (Fig. 3; E1). In pleomorphic adenomas, the CNN models concentrated on the interstitial connective tissue. (Fig. 4; E2, E3, E5, A2, A3)

## Discussion

### Binary classification of benign and malignant salivary gland tumors

With regard to the binary differential diagnosis between the two benign salivary gland tumors - combined into one category - and the four malignant tumors summarized as one category, both CNN and Vision Transformer architectures achieved compelling results. The balanced accuracy ranged from 91.29% for ResNet50 to 100% for Xception. The F1 score ranged from 0.8980 for ResNet50 for the detection of malignant tissue to 1.0 for both tasks for Xception. Considering the corresponding confusion matrices (Figure S7), it is remarkable that VGG19 and Xception never misclassified malignant tissue as benign (Negative Predictive Value (NPV) for the diagnosis of malignant tissue: 100%). Inception-ResNet-v2 performed worst in this respect, misclassifying malignant tissue as benign in 214 out of 100,840 individual tiles (Figure S7). Additionally, false positive findings for malignant tissue occurred least frequently when using Xception (only in 4 cases out of 131,432). This type of confusion occurred most frequently for ResNet50 (22,898 out of 131,432). In Summary, VGG19 and Xception demonstrate high NPVs for the diagnosis of malignant salivary gland tumors, making them effective screening tools for malignancy. This could support pathologists in rapidly identifying malignancies and prioritizing cases for further immunohistochemical analysis.

### Subclassification of benign salivary gland tumors and normal salivary gland tissue

In the subclassification of benign salivary gland tumors (Warthin tumor and pleomorphic adenoma) compared to normal salivary gland tissue, the CNN-models and the ViT achieved promising results with a balanced accuracy ranging from 0.9172 (Inception-ResNet-v2) to 0.9557 (Xception). The F1 score fluctuated from 0.7096 (Inception-ResNet-v2) in the detection of normal salivary gland tissue to 0.9848 (Xception) in the identification of pleomorphic adenoma. The confusion matrices reveal similar classification errors, indicating similar recognition patterns between the applied CNNs. Despite these errors, the applied CNNs achieve high accuracy and F1 scores, distinguishing between benign tumor and normal salivary gland tissue. Perspectively, with further training on larger datasets, the models could become a useful and supportive tool for the histopathological diagnosis of benign salivary gland tumors.

Of note, the accurate subclassification of benign salivary gland tumors is clinically and prognostically relevant as pleomorphic adenoma shows a higher probability of recurrence (2,9 − 6,7%) and malignant transformation in recurrence (3% in recurrence) in comparison to the Warthin tumor [2].

### Classification of malignant and normal tissue and subclassification of malignant tissues

The differentiation of malignant salivary gland tumor entities is challenging in histopathological practice. When distinguishing between squamous cell carcinoma, acinic cell carcinoma, adenoid cystic carcinoma, mucoepidermoid carcinoma and normal salivary gland tissue, the results were less favorable than those observed in the initial two tasks. In this sense, the balanced accuracy ranged from 0.5645 for Vision Transformer to 0.7151 for Inception-ResNet-v2. The minimum F1 score was 0.1689 for the Vision Transformer in the detection of squamous cell carcinoma, while the maximum F1 score achieved was 0.7859 for the Inception-ResNet-v2 in the recognition of mucoepidermoid carcinoma. A frequent similar confusion in the classification of malignant salivary gland tumors was observed in all models, indicating similar pattern recognition.

### Class activation maps

CAMs and attention maps, as tools of explainable Artificial Intelligence were applied and analysed to ensure that CNN and ViTs decision processes are based on biologically and histopathologically relevant features [16]. In this sense, they could help pathologists to identify and focus on the areas that the network deems most relevant for predicting a given class.

For the correct classification of benign tumors, CNNs mainly focused on interstitial connective tissue (Fig. 1; B5, D5, E5). In regions with a high nucleus density, ResNet50 focused on the cytoplasm (Fig. 1; B1 and B2). For the correct classification of malignant tissue, ConvNeXt (as illustrated in Fig. 2; E3 and E5) and Vision Transformer (as shown in Fig. 2: F4 and F5) prioritized the cell nuclei. For the classification of Warthin tumor, ViT (Fig. 3; F2, F3) and ConvNext (Fig. 3; E3, E5) primarily focused on the cell-rich lymphoid stroma. Additionally, ConvNext also weighted the characteristic oncocytic epithelium of the Warthin tumor (Image E1 in Fig. 3 ) [2]. Considering pleomorphic adenoma, CNN models focused on interstitial connective tissue, as shown by the highlighted connective tissue in Fig. 4 E2, E3, E5 or in Fig. 4 A2, A3.

Taken together, CNNs and Vision Transformers identify histopathologically relevant patterns for specific classification tasks. Of note, for all entities the applied models occasionally relied on non-traceable, respectively random structures. Class Activation Maps revealed obscure weightings, demonstrating that while CNNs can identify comprehensible patterns for specific tasks, some classifications were based on unclear patterns despite accurate diagnoses. Regarding radiological procedures such as MRI [17], CT [18] or sonography [19], previous research has demonstrated that the integration of deep learning models can enhance preoperative diagnosis. However, postoperative histopathological analysis represents the gold standard for diagnosing salivary gland tumors [20].

Only a few histopathological studies have explored the CNN based automated histopathological diagnosis of salivary gland tumors. Schulz et al. [21] investigated the impact of CNNs differentiating grouped malignant salivary gland tumors as one category (including adenoid cystic carcinoma, adenocarcinoma not other specified, acinic cell carcinoma, basal cell carcinoma, mucoepidermoid carcinoma, and myoepithelial carcinoma) from adipose tissue, connective tissue, and background. They achieved accuracies between 18.8% and 84.7% and F1-scores ranging from 0.07 to 0.72. In contrast, the presented approach examined grouped malignant tumors into a single category against grouped benign tumors, attaining higher F1-scores (0.8980 for ResNet50 to 1.0 for Xception). However, Schulz et al. categorized tissues into four classes and used significantly less individual tiles (83,819). Li et al. [22] investigated the potential of a ViT for the differential diagnosis of six benign (pleomorphic adenoma, Warthin tumor, myoepithelioma, basal cell adenoma, oncocytoma, cystadenoma) and two malignant tumor entities (mucoepidermoid carcinoma, adenoid cystic carcinoma) as well as normal salivary gland tissue as a control. By using a modified ViT, Li et al. achieved F1 scores, recall, and precision values of 0.9848 each, comparable to our binary classification (up to 1.0 for Xception). For the subclassification of malignant tumors, our F1 scores (0.1689 to 0.7859) were lower, likely due to Li et al.‘s larger dataset (3,046 WSIs from 978 patients vs. 335 WSIs from 131 patients). The preprint study of Alsanie et al. [23] assessed the differential diagnosis of two benign tumor entities (pleomorphic adenoma, basal cell adenoma) versus four malignant tumors (mucoepidermoid carcinoma, adenoid cystic carcinoma, acinic cell carcinoma and carcinoma ex pleomorphic adenoma). Similar to our approach, they grouped benign and malignant tumors separately, therefore using QuPath and random forest classifiers (RF) alongside CNN models (ResNet18, ResNet50, Efficient-NetB0, Efficient-NetB3). The QuPath RF achieved an F1 score of 0.9, and the customized RF reached 0.95, comparable to our Xception results. Their CNN models performed slightly worse, with F1 scores ranging from 0.81 to 0.87. When subtyping malignant tumors using 120 WSIs without any surrounding benign tissue, the RF models achieved higher F1 scores (0.92 and 0.95) compared to our models (up to 0.79). This discrepancy results from a larger dataset of malignant tumors (120 WSIs vs. 87 WSIs) and the additional inclusion of normal salivary gland tissue in our approach. The inclusion of normal salivary gland tissue in our subtyping may have resulted in a slight increase in external validity, as histopathological slides of salivary gland tumors often contain a substantial proportion of normal salivary gland tissue.

The presented study has several limitations. Model training and evaluation were exclusively based on standard H&E-stained histological sections. While H&E-stained sections allow reliable diagnosis of typical salivary gland tumors, diagnostically challenging cases sometimes require immunohistochemical analysis, molecular testing or mutation screening, which were not addressed in the present approach [2]. Accordingly, future studies may investigate whether the integration of immunohistochemical markers, molecular profiling, or mutation analyses can improve model performance, particularly in diagnostically challenging cases.

Due to the rarity of malignant salivary gland tumors, a retrospective approach had to be followed. Therefore, histological slides had to be selected over a time interval of 20 years, leading to variations in staining intensities. This issue had to be addressed by normalization processes, as previously described [6]. The limited sample size of 19 to 26 WSIs per malignant entity represented a challenge for model training and testing. Additionally, the histopathological heterogeneity of some entities, such as acinic cell carcinoma (solid, follicular, microcystic, or papillary-cystic patterns) and adenoid cystic carcinoma (cribriform, tubular, or solid patterns), suggests that not all growth patterns were adequately represented [2]. This histopathological variety could be accountable for the suboptimal performance of the models in the sub-classification of malignant tumors. While including the most prevalent diagnoses with 2 benign and 4 malignant entities, rarer entities were not considered. However, infrequent entities pose a particular diagnostic challenge. For training on rare entities and respecting the histopathologic diversity within one entity, cross-regional collaborations could ensure a sufficient variety and volume of data.

## Conclusion

The models demonstrated strong performance in distinguishing benign from malignant salivary gland tumors, particularly with the high NPV of the Xception model, and showed promise in differentiating benign tumors from each other and from normal tissue. These findings suggest the applicability of CNNs and ViT for supporting rapid postoperative pathological screening on H&E-stained slides. These findings suggest the potential applicability of CNNs and ViT for supporting rapid binary benign-malignant screening on H&E-stained slides in the postoperative setting. Nevertheless, expanding the spectrum of tumor entities through inter-institutional collaboration will be essential to fully realize the clinical applicability of this approach. However, given the limited number of malignant cases, the restricted spectrum of tumor entities, and the observed misclassifications in malignant subtyping, further validation on larger, multi-institutional datasets —potentially incorporating immunohistochemical and molecular analyses— is required before clinical implementation can be considered.

## Electronic Supplementary Material

Below is the link to the electronic supplementary material.

Supplementary Material 1

## References

[1] Alsanie I, Rajab S, Cottom H, Adegun O, Agarwal R, Jay A et al (2022) Distribution and Frequency of Salivary Gland Tumours: An International Multicenter Study. Head Neck Pathol 16:1043–1054. 10.1007/s12105-022-01459-0 35622296 10.1007/s12105-022-01459-0 PMC9729635

[2] WHO Classification of Tumours Editorial Board (2022) Head and neck tumours. Lyon (France): International Agency for Research on Cancer. (WHO classification of tumours series, 5th ed.; vol. 9) https://publications.iarc.fr/

[3] Guntinas-Lichius O, Beck-Broichsitter B, Füreder T, Haderlein M, Ihrler S, Kissinger G, Klinghammer K, Klußmann JP, Kocher F, Mach N, Meyer MF, Münter M, Olivier T, Schafhausen P, Vogl TJ, Wollenberg B (2024) Recommendations from the society for diagnosis and therapy of haematological and oncological diseases, Salivary Gland Carcinomas. ONKOPEDIA. https://www.onkopedia-guidelines.info/s/4AB6EZ. Accessed 28 Nov 2024

[4] van Herpen C, Vander Poorten V, Skalova A, Terhaard C, Maroldi R, van Engen A et al (2022) Salivary gland cancer: ESMO-European Reference Network on Rare Adult Solid Cancers (EURACAN) Clinical Practice Guideline for diagnosis, treatment and follow-up. ESMO Open 7:100602. 10.1016/j.esmoop.2022.100602 36567082 10.1016/j.esmoop.2022.100602 PMC9808465

[5] Bankhead P et al (2017) QuPath: Open source software for digital pathology image analysis. Sci Rep. 10.1038/s41598-017-17204-5 29203879 10.1038/s41598-017-17204-5 PMC5715110

[6] Reinhard E, Adhikhmin M, Gooch B, Shirley P (2001) Color transfer between images. IEEE Comput Graph Appl 21:34–41. 10.1109/38.946629

[7] Simonyan K, Zisserman A (2015) Very deep convolutional networks for large-scale image recognition 10.48550/arXiv.1409.1556

[8] He K, Zhang X, Ren S, Sun J (2016) Deep residual learning for image recognition (2016). IEEE Conf. Comput. Vis. Pattern Recognit. CVPR, Las Vegas, NV, USA: IEEE, pp. 770–8. 10.1109/CVPR.2016.90

[9] Szegedy C, Ioffe S, Vanhoucke V, Alemi A (2017) Inception-v4, Inception-ResNet and the impact of residual connections on learning. Proc AAAI Conf Artif Intell 31. 10.1609/aaai.v31i1.11231

[10] Chollet F, Xception (2017) Deep Learning with Depthwise Separable Convolutions. IEEE Conf. Comput. Vis. Pattern Recognit. CVPR, Honolulu, HI: IEEE; 2017, pp. 1800–7. 10.1109/CVPR.2017.195

[11] Liu Z, Mao H, Wu C-Y, Feichtenhofer C, Darrell T, Xie S. A ConvNet for the 2020s 2022. 10.48550/arXiv.2201.03545

[12] Dosovitskiy A, Beyer L, Kolesnikov A, Weissenborn D, Zhai X, Unterthiner T et al (2021) An image is worth 16x16 words: transformers for image recognition at scale. 10.48550/arXiv.2010.11929

[13] Abadi M, Agarwal A, Barham P, Brevdo E, Chen Z, Citro C et al (2016) TensorFlow: Large-Scale Mach Learn Heterogen Distrib Syst. 10.48550/arXiv.1603.04467

[14] Sokolova M, Lapalme G (2009) A systematic analysis of performance measures for classification tasks. Inf Process Manag 45:427–437. 10.1016/j.ipm.2009.03.002

[15] Zhou B, Khosla A, Lapedriza A, Oliva A, Torralba A (2015) Learning deep features for discriminative localization. 10.48550/arXiv.1512.04150

[16] Huff DT, Weisman AJ, Jeraj R (2021) Interpretation and visualization techniques for deep learning models in medical imaging. Phys Med Biol 66(TR01):04. 10.1088/1361-6560/abcd17 10.1088/1361-6560/abcd17 PMC8236074 33227719

[17] Xia X, Feng B, Wang J, Hua Q, Yang Y, Sheng L et al (2021) Deep Learning for Differentiating Benign From Malignant Parotid Lesions on MR Images. Front Oncol 11:632104. 10.3389/fonc.2021.632104 34249680 10.3389/fonc.2021.632104 PMC8262843

[18] Zhang H, Lai H, Wang Y, Lv X, Hong Y, Peng J et al (2021) Research on the Classification of Benign and Malignant Parotid Tumors Based on Transfer Learning and a Convolutional Neural Network. IEEE Access 9:40360–40371. 10.1109/ACCESS.2021.3064752

[19] Zhang G, Zhu L, Huang R, Xu Y, Lu X, Chen Y et al (2023) A deep learning model for the differential diagnosis of benign and malignant salivary gland tumors based on ultrasound imaging and clinical data. Quant Imaging Med Surg 13:2989–3000. 10.21037/qims-22-950 37179911 10.21037/qims-22-950 PMC10167466

[20] Khan S, Nair NG (2022) Diagnostic Accuracy of FNAC and Ultrasonography in Salivary Gland Lesions in Comparison with Histopathology. J Clin Diagn Res. 10.7860/JCDR/2022/57726.17109

[21] Schulz T, Becker C, Kayser G (2023) Ein Vergleich von 4 konvolutionalen neuronalen Netzen in der histopathologischen Diagnostik von Speicheldrüsenkarzinomen. HNO 71:170–176. 10.1007/s00106-023-01276-z 36734999 10.1007/s00106-023-01276-z PMC9950222

[22] Li M, Shen Z, Xian H, Zheng Z, Yu Z, Liang X et al (2024) A recognition system for diagnosing salivary gland neoplasms based on vision transformer. Am J Pathol S0002944024003961. 10.1016/j.ajpath.2024.09.010 10.1016/j.ajpath.2024.09.010 39490441

[23] Alsanie I, Shephard A, Azarmehr N, Rajpoot N, Khurram SA (2022) Using artificial intelligence for analysis of histological and morphological diversity in salivary gland tumors. 10.21203/rs.3.rs-1966782/v1

