# Supplementary material for: Deep learning-based histopathological classification and subclassification of benign and malignant salivary gland tumors
Source: Eur Arch Otorhinolaryngol. 2026 Mar 5;283(6):3905–15. doi: 10.1007/s00405-026-10082-6 (PMC13249714; doi:10.1007/s00405-026-10082-6)
Supplement: Supplementary file 1 — Supplementary Material 1 [file 405_2026_10082_MOESM1_ESM.docx]

**Supplements**


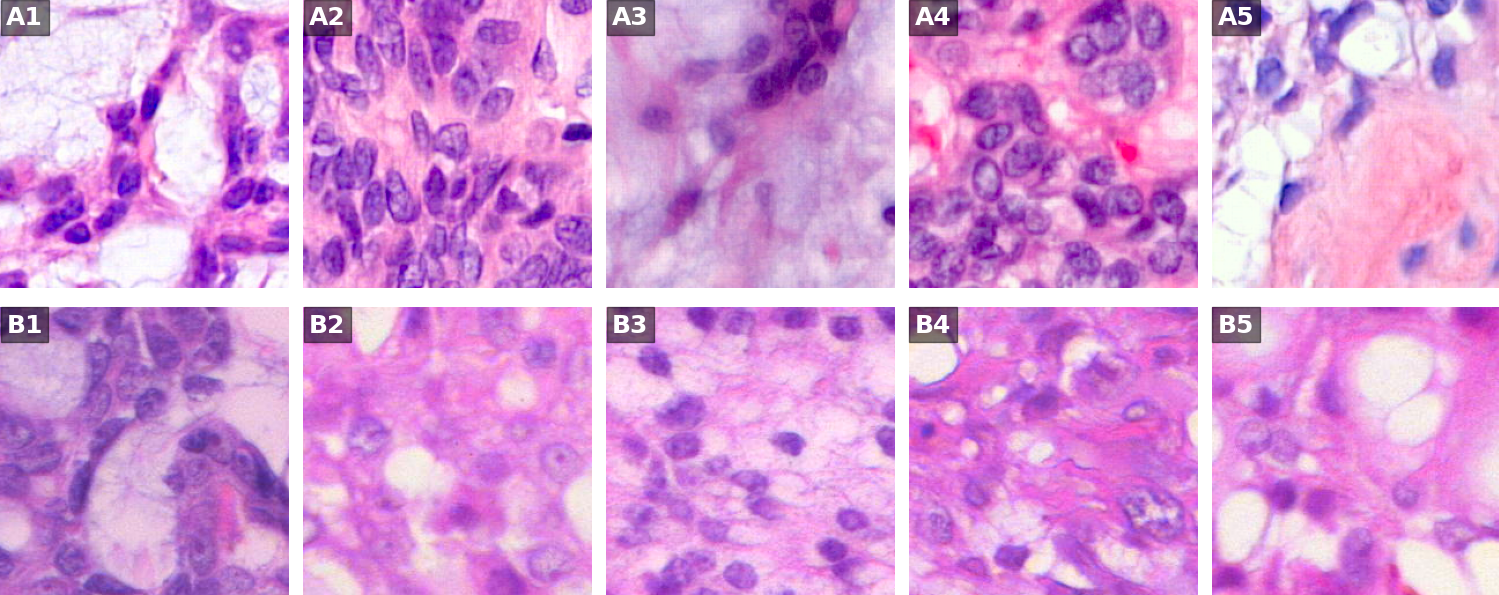


*Figure S1: Exemplary tiles for tissue classes “benign” (A1-A5) and “malignant” (B1-B5).*


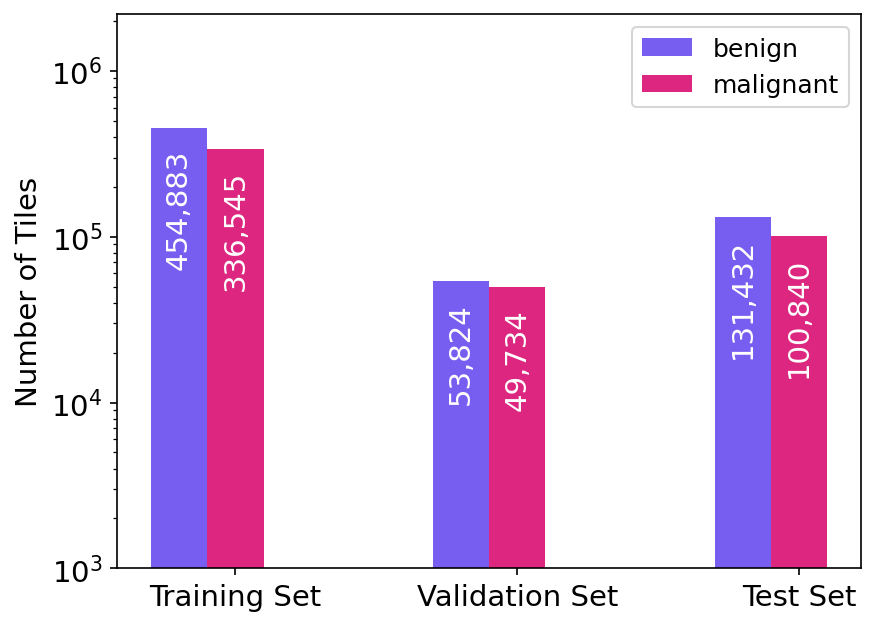


*Figure S2: Class distribution on a logarithmic scale for “benign” and “malignant” in the training, validation and test set.*


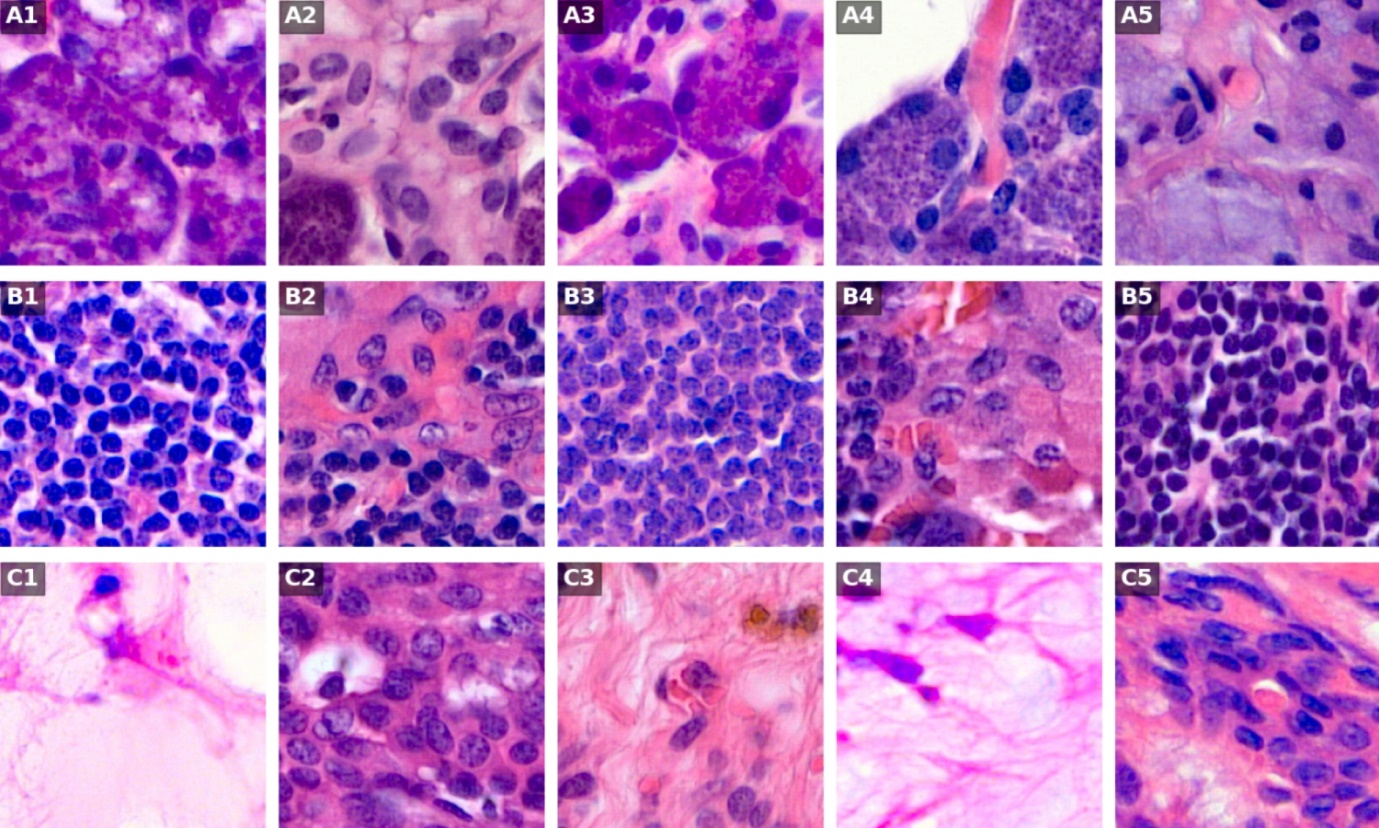


*Figure S3: Exemplary tiles for benign tissue classes “salivary gland” (A1-A5), “Warthin tumor”(B1-B5) and “pleomorphic adenoma” (C1-C2).*


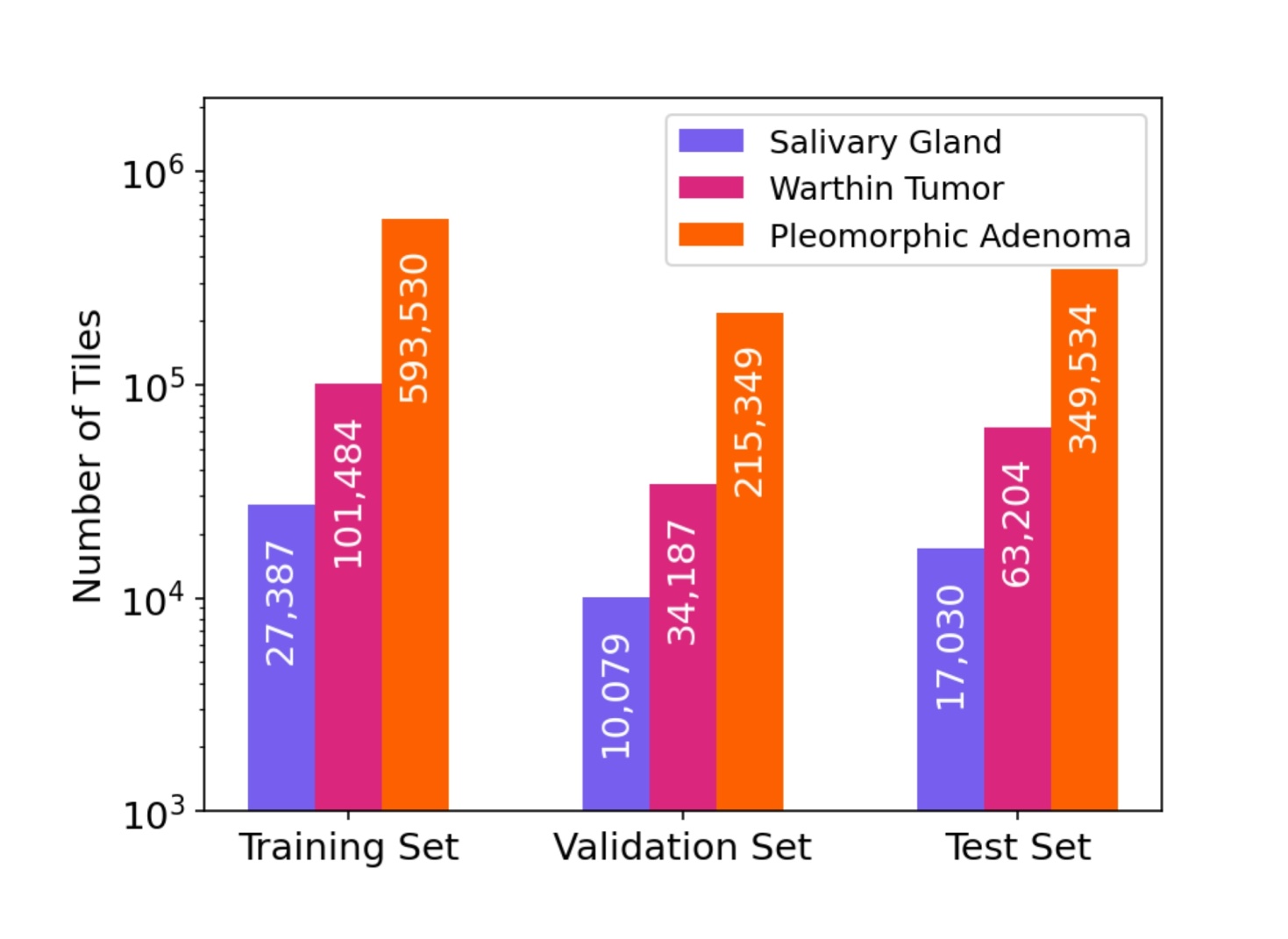


*Figure S4: Class distribution on a logarithmic scale for salivary gland, Warthin tumor and pleomorphic adenoma in the training, validation and test set.*


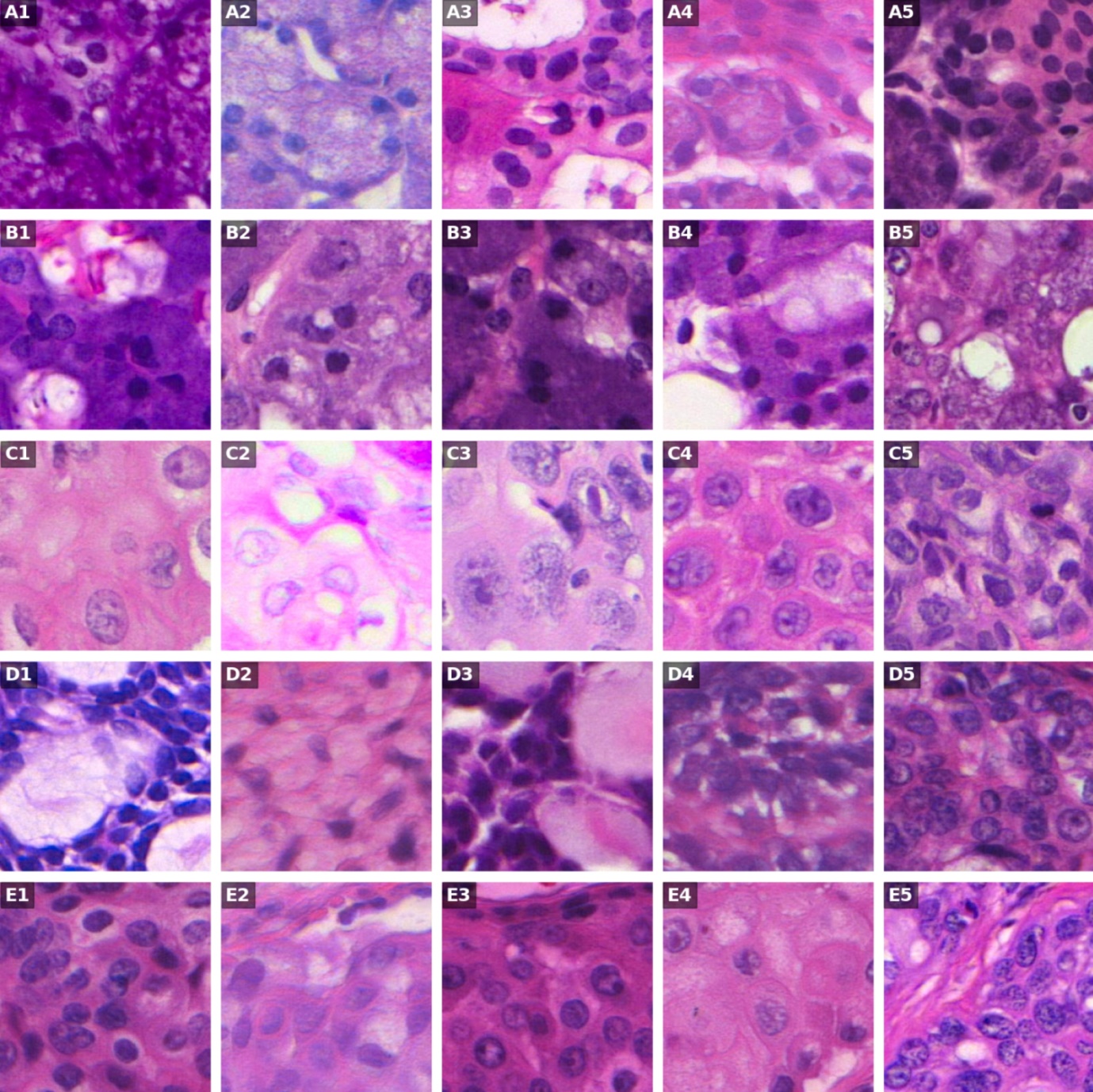


*Figure S5: Exemplary tiles for “salivary gland tissue”(A1-A5) and malignant tissue classes “acinic cell carcinoma” (B1-B5), “squamous cell carcinoma” (C1-C2), “adenoid cystic carcinoma” (D1-D5) and “mucoepidermoid carcinoma” (E1-E5).*


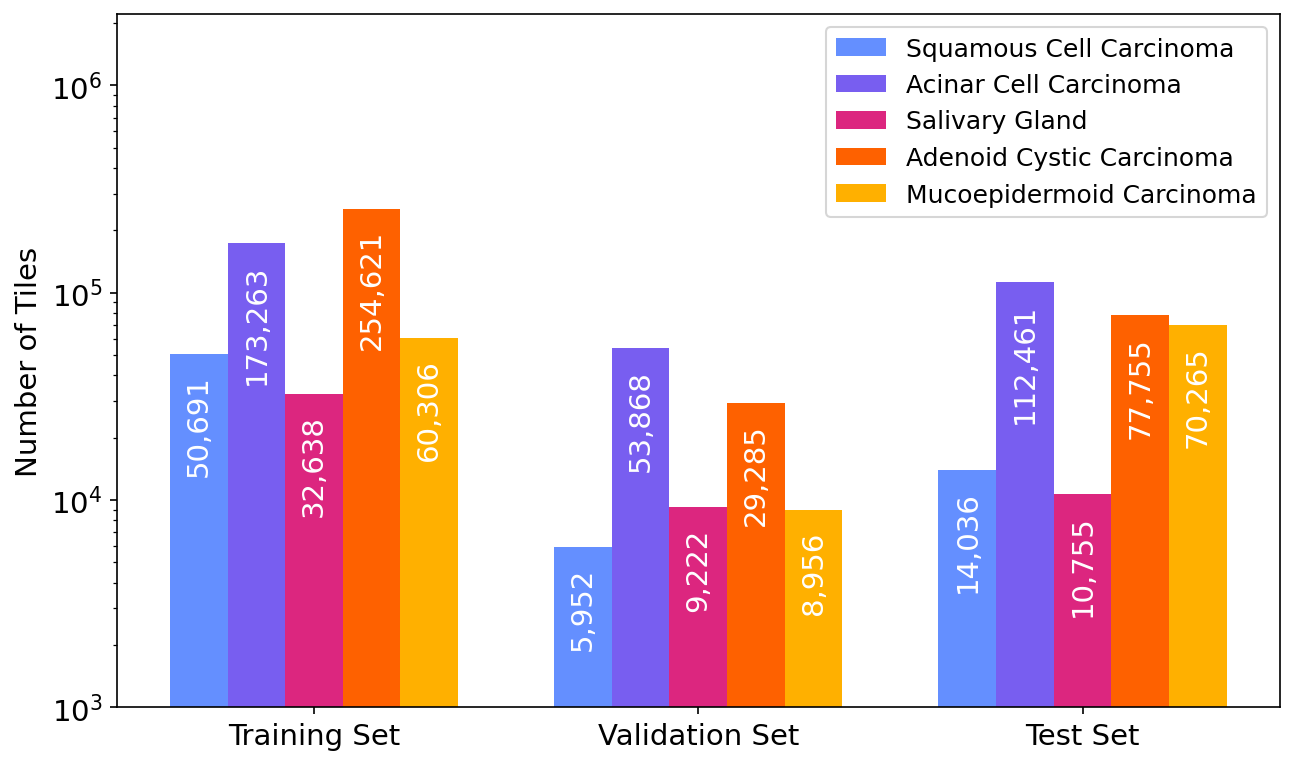


*Figure S6: Class distribution on a logarithmic scale for squamous cell carcinoma, acinic cell carcinoma, salivary gland, adenoid cystic carcinoma and mucoepidermoid carcinoma in the training, validation and test set.*

*
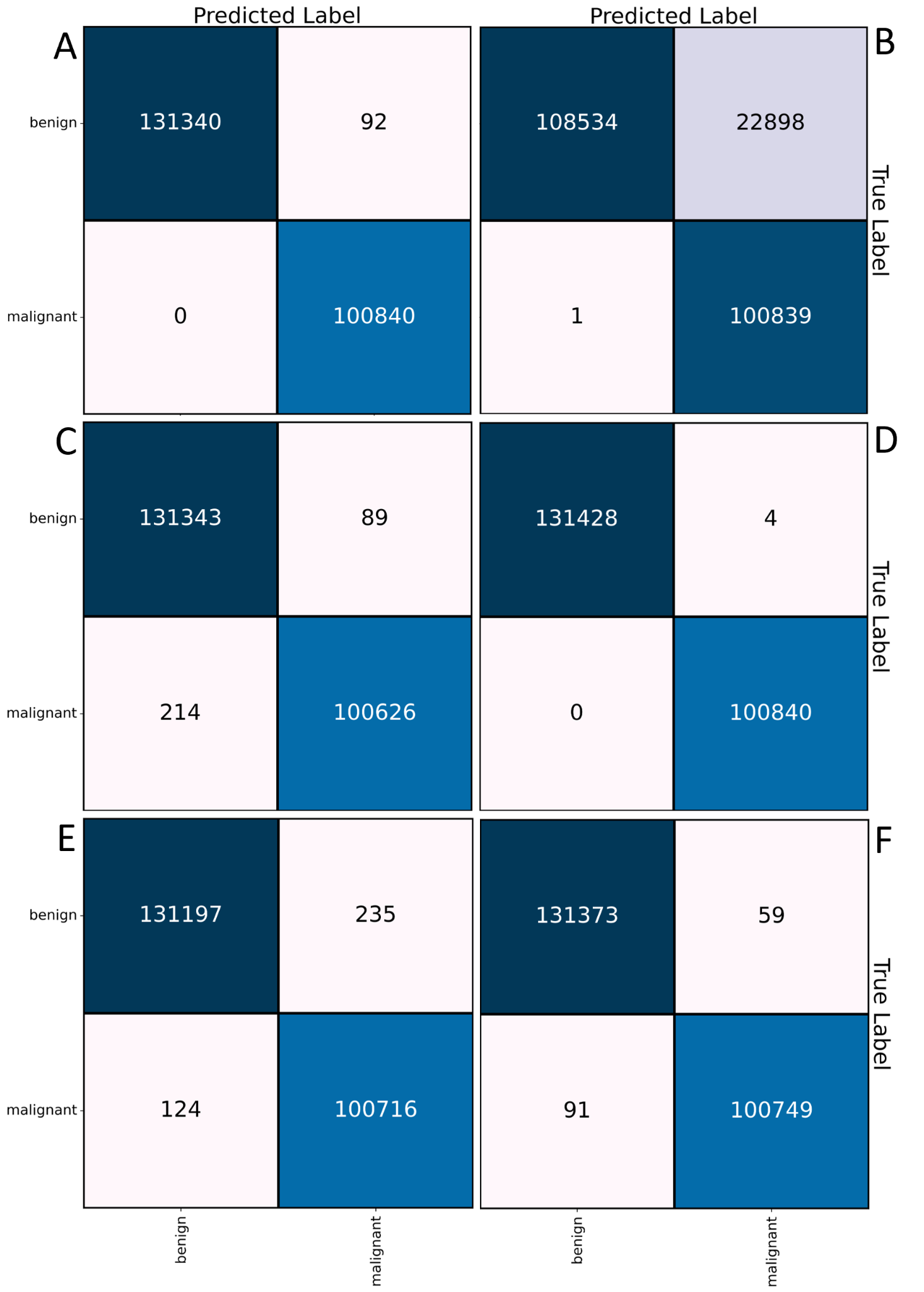
*

*Figure S7: Confusion matrices for VGG19 (A), ResNet50 (B), Inception-ResNet-v2 (C), Xception (D), ConvNeXt (E) and ViT (F) for classification of benign and malignant tumors on the test set.*

*
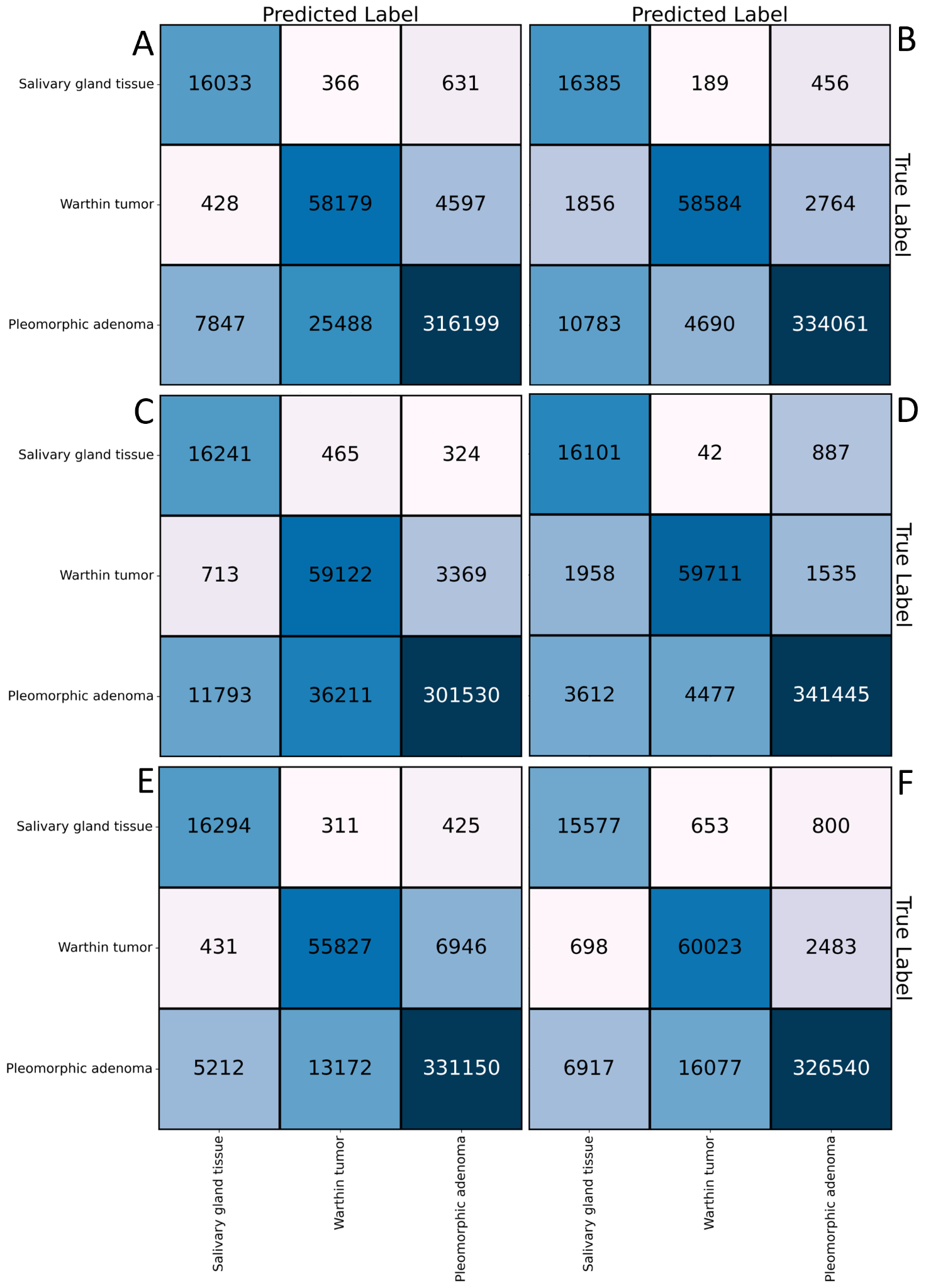
*

*Figure S8: Confusion matrices for VGG19 (A), ResNet50 (B), Inception-ResNet-v2 (C), Xception (D), ConvNeXt (E) and ViT (F) for subclassification of benign tissue on the test set.*

*
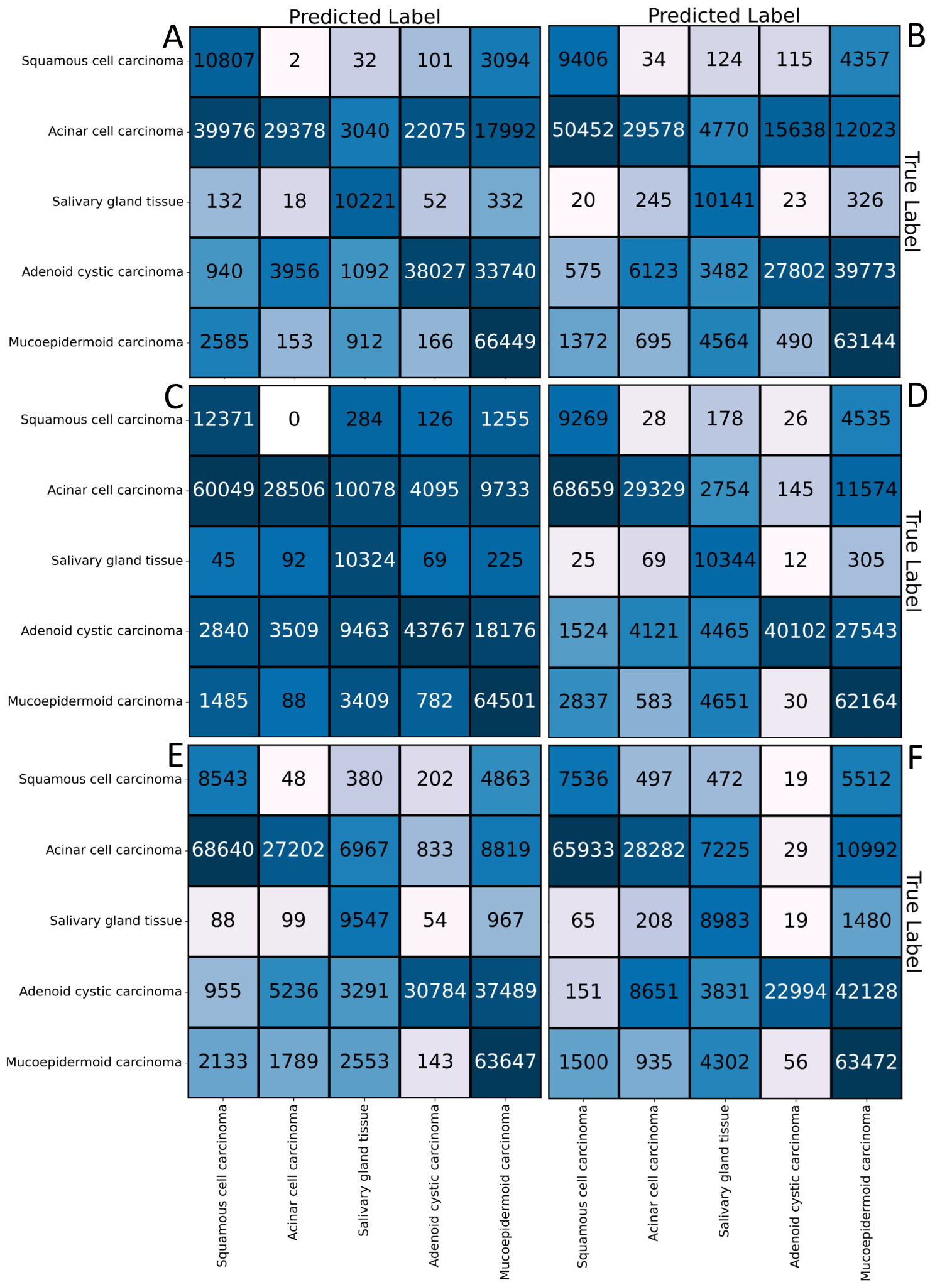
*

*Figure S9: Confusion matrices for VGG19 (A), ResNet50 (B), Inception-ResNet-v2 (C), Xception (D), ConvNeXt (E) and ViT (F) for subclassification of malignant tissue on the test set.*
